# An Updated Review and a Case Report: Stress Ulcer Prophylaxis

**DOI:** 10.7759/cureus.60811

**Published:** 2024-05-21

**Authors:** Joy L Zhao, Christina Tofani, Anthony Infantolino

**Affiliations:** 1 Department of Gastroenterology and Hepatology, Sidney Kimmel Medical College, Philadelphia, USA; 2 Division of Gastroenterology and Hepatology, Cooper University Hospital, Mount Laurel, USA

**Keywords:** case report, review, prophylaxis, stomach ulcer, peptic ulcer

## Abstract

After encountering a unique patient case, we revisit the updated literature on stress ulcer prophylaxis with more updated studies. A 47-year-old male came to the hospital and was found to have acute cholecystitis. After undergoing urgent cholecystectomy, the patient developed melena and a 6 mg/dL drop from 12.5 g/dL to 6.5 g/dL in hemoglobin. He was found to have a gastric ulcer and was started on a proton pump inhibitor, which posed the question of whether or not stress ulcer prophylaxis was indicated. Therefore, the pathophysiology of stress ulcer prophylaxis is refreshed, discussing the various mechanisms through which stress ulcers form in a clinical context. Then, the main risk factors and indications for stress ulcer prophylaxis are defined based on current literature, further investigating whether or not stress ulcer prophylaxis has shown benefit and protection in various patient groups. Additionally, this review discusses the adverse effects of stress ulcer prophylaxis, including dysbiosis, community-acquired pneumonia, nutritional deficiencies, drug interactions, and fractures. Finally, inappropriate stress ulcer prophylaxis and contributing factors to overutilization are discussed, and alternative approaches to prevent stress ulcer formation are covered, including early enteral nutrition. Overall, there are mixed conclusions on the effectiveness of stress ulcer prophylaxis in noncritical patients. There are many adverse effects and unnecessary costs associated with inappropriate administration, and many studies have found that it should be reserved for specific clinical indications.

## Introduction

For background, stress ulcers have been an area of controversy for many years. They can present with abdominal pain in addition to having evidence of an upper gastrointestinal bleed. More specifically, this is when there is vomiting of blood, known as hematemesis, or dark, tarry, black stool, known as melena. Depending on the severity of the stress ulcer, there may also be changes in the patient's hemoglobin, also known as blood levels, as there are varying degrees of bleeding. This subsequently can cause changes in the patient's hemodynamics. 

Stress ulcers can develop after events such as shock, sepsis, burns, and trauma and typically occur in hospitalized patients. Early stress ulcers generally form in the proximal stomach within hours. Few ulcers eventually lead to clinical bleeding. Late stress ulcers, which develop after multiple days of hospitalization, are more distal in the stomach and erode deeper into the gastric mucosa [1,2].

The pathophysiology in the clinical scenarios mentioned before involves luminal acid and ischemia, which is inadequate blood supply. Additionally, gastric mucosal barrier dysfunction contributes to stress ulcers [3]. More specifically, shock and sepsis can lead to increases in uremic toxins, increased bile salt reflux, and compromised blood flow, which ultimately breaks down the stomach’s protective glycoprotein mucous layer [4,5]. Similarly, the actual production of the protective stomach mucosa is decreased in the setting of shock, sepsis, and trauma [6,7]. Another potential physiological mechanism leading to stress ulcers is acid hypersecretion due to excess gastrin hormone stimulation from parietal cells, which are the stomach's epithelial cells [8,9]. This has been noted in particular with patients who have had traumatic head injuries.

Stress ulcer prophylaxis (SUP) is indicated in certain patients as defined by the American Society of Health System Pharmacists (ASHP), requiring at least one major risk factor or two minor risk factors. This patient population is defined as patients admitted to an ICU with coagulopathy, multiple traumas, >48 hours of mechanical ventilation, gastrointestinal bleeding (GIB) within the past year, sepsis, hepatic or renal transplant, > one week in the ICU, occult GIB for >6 days, >250 mg of steroid therapy daily, to name a few risk factors [10]. However, many studies have highlighted that the main risk factors for significant stress ulcer bleeding are mechanical ventilation for over 48 hours and coagulopathies, including platelets less than 50x109/L, international normalized ratio (INR) over 1.5, or partial thromboplastin time (PTT) over two times the control [5,11-15]. Potential additional conditions associated with stress ulcers are a previous history of peptic ulcer disease, gastroesophageal reflux disease, chronic nonsteroidal anti-inflammatory drug use, Zollinger-Ellison syndrome, or brain or spinal cord injury [16].

This case led to the debate of whether or not SUP was indicated in this clinical scenario, so we performed an updated review of the recent literature involving SUP regarding use, adherence, adverse effects, and cost.

## Case presentation

A male in his mid-40s with a history of type II diabetes, obesity, poorly controlled coronary artery disease, peripheral vascular disease, and hypertension is admitted to the hospital with right upper quadrant abdominal (RUQ) pain, fever, and chills. His home medications include lisinopril, simvastatin, metformin, and baby aspirin. Abdominal ultrasound revealed a thick-walled gallbladder with stones and pericholecystic fluid but no biliary ductal dilation. Unfortunately, since this was an old case, no ultrasound image was available upon chart review. Labs were notable for leukocytosis with left shift, hyperbilirubinemia (total bilirubin of 5.6 mg/dL and direct bilirubin of 4.4 mg/dL), and elevated transaminases (aspartate transaminase (AST) of 215 mg/dL and alanine transaminase (ALT)of 290 mg/dL). The coagulation studies were normal. The patient was diagnosed with acute cholecystitis and observed for 24 hours, but he continued to have severe pain. He subsequently underwent an urgent cholecystectomy. On postoperative day 3, the patient reported melena. Stat labs were drawn and were notable for hemoglobin of 10.0 g/dL from hemoglobin of 12.5 g/dL on admission. A second 20-gauge IV was placed, and the surgery and gastroenterology teams were notified. After further melena, the patient's blood pressure was 80/50 mmHg. Repeat hemoglobin was 6.5 g/dL, so the patient was transfused with two units of cross-matched packed red blood cells. Based on the patient's history and presentation, a drug-induced ulcer was felt to be less likely. He was transferred to the ICU for monitoring and urgent endoscopy. He was given pantoprazole 80 mg IV bolus, followed by pantoprazole IV infusion. He was intubated for the endoscopic procedure for airway protection. An endoscopy revealed a 1.0-1.5 cm ulcer with a visible vessel in the distal gastric antrum actively bleeding. The rest of the gastric mucosa and gastroesophageal junction were normal, and Helicobacter pylori testing was negative. The ulcer was treated with 2 cc of 1:10,000 epinephrine injection and bipolar electrocautery. The bleeding appeared to have stopped, and he was extubated post procedure. The patient’s hemoglobin remained stable at 8 g/dL and was placed on PPI twice daily with no further signs of recurrent bleeding (Table 1).

**Table 1:** Patient Laboratory Values

Test Name	Patient Value	Reference Range
Total Bilirubin	5.6 mg/dL	0.2 and 1.3 mg/dL
Direct Bilirubin	4.4 mg/dL	<0.3 mg/dL
Aspartate Transferase	215 mg/dL	<40 U/L
Alanine Transaminase	290 mg/dL	<40 U/L
Admission Hemoglobin	12.5 g/dL	14-18 g/dL
Repeat Hemoglobin 1	10.0 g/dL	14-18 g/dL
Repeat Hemoglobin 2	6.5 g/dL	14-18 g/dL
Final Hemoglobin	8 g/dL	14-18 g/dL

## Discussion

In terms of stress ulcer complications, patients who are not critically ill have a stress ulcer bleeding rate of <1% [4,17,18], while in critically ill patients, the rate of ulcer-related bleeding varies from 5-25% [4]. In some studies, non-ICU patients who received SUP experienced a slight decrease in GIB [17,19,20]. A subgroup analysis found significant reductions in clinically significant bleeding with SUP in neurosurgical patients but not in surgical or medical ICU patients with risk factors [21].

Furthermore, studies concluded that neither proton-pump inhibitors nor histamine 2 receptor antagonists significantly reduced bleeding in patients with low to moderate GIB risk [22]. Similarly, hemodynamically stable patients on anticoagulation failed to have lower rates of stress ulcers when on acid-suppressive therapy [18]. Ogasawara et al. evaluated the rate of SUP administration before and after establishing a prophylaxis criteria checklist. While the incidence of GIB before and after implementing the checklist remained to be 4%, the proportion of SUP administration decreased 62%, demonstrating no increase in GIB with a decrease in SUP [23].

Whether or not there is a decreased risk of GIB with SUP, the number needed to treat (NNT) to prevent one nosocomial GIB was 770, reflecting the questionable requirement for unnecessary prophylaxis [11,12,17]. Therefore, it is important to highlight that routine SUP in the clinical setting is not indicated in most patients because of insignificant benefits, added-on costs, and risks of adverse effects such as infection, nutritional deficiency, fractures, and reduced drug efficacy.

Specifically, SUP decreases the acidity of the stomach, potentially leading to impaired destruction of microorganisms, which therefore culminates in bacterial overgrowth [24]. Additionally, SUP can cause patients to develop dysbiosis, where the beneficial gastrointestinal microbe signaling is lost. This paves the way for pro-inflammatory cytokine release and immune dysregulation [25]. Particularly, prophylaxis can make patients susceptible to Clostridium difficile, community-acquired pneumonia from microaspiration, or spontaneous bacterial peritonitis in cirrhotic patients [20,24,26]. These side effects are associated with higher mortality, prolonged hospitalization, and increased cost [25]. On the contrary, a recent meta-analysis of randomized control trials found no significant difference in the incidence of pneumonia, C. difficile, or mortality in patients on SUP [21,27].

Additionally, PPIs competitively inhibit cytochrome CYP2C19, leading to a potential impact on the efficacy of certain drugs [10].

In terms of nutritional deficiency, the previously mentioned bacterial overgrowth can lead to overconsumption of cobalamin, placing patients at risk for vitamin B12 deficiency. Similarly, by the same mechanism of bacterial overgrowth and consumption, there is reduced iron and magnesium absorption [24,28].

The long-term use of proton pump inhibitors (PPIs) may also lead to a slightly increased risk of fracture [29]. The possible theories to explain this association are increased secretions of histamine and secondary hyperparathyroidism because of hypergastrinemia [30]. Hypergastrinemia can occur from prolonged PPI because of parietal cell hypertrophy and enterochromaffin-like cell hyperplasia through rebound acid hypersecretion [24]. Moreover, there is potential for decreased calcium absorption and direct inhibition of osteoclasts in bone resorption from PPI, increasing the risk of osteoporosis [31].

There is an overall lack of clear benefits of SUP in non-ICU patients. This is reflected in excess spending as patients are given unindicated SUP, which should be limited to patients at high risk for clinical bleeding [32]. A retrospective study found that 54% of non-ICU patients were discharged home on anti-secretory therapy, despite none of them meeting evidence-based criteria for SUP: a very unnecessary additional expenditure from SUP overutilization [33]. In parallel, a separate study found that 51.9% of patients who downgraded from the ICU were maintained on SUP, even though there were no indications for its use, increasing risks that were previously mentioned [34-36].

SUP overuse could be in part because of a lack of awareness of clinical guidelines as a multicenter study in China found that 46% of their surgical and perioperative physicians were unaware of their SUP guidelines [37]. Similarly, an observational study of an inpatient surgical ward found that 48% of SUP did not follow ASHP criteria and was therefore an excess cost [22,38]. Similarly, a prospective study in Palestine found that only 16.7% of patients were appropriately prescribed SUP according to ASHP guidelines, a suboptimal practice that was comparably found in Iran [39,40].

To better address potential GIB in the hospital setting, early enteral nutrition has been found to be protective, and it avoids dysbiosis in the gut [25]. More specifically, changing SUP from intravenous to enteral within 72 hours has been found to be a safer and more cost-effective SUP method, which can be further studied as an alternative to current practices [22,41].

## Conclusions

In conclusion, there are mixed studies on the effectiveness of SUP in non-ICU patients. Many studies have found that SUP should be reserved for patients with specific indications, that is, ventilated patients for more than 48 hours with associated coagulopathies. Nevertheless, it has been shown that there are many complications and costs associated with inappropriate SUP administration, which needs to be further protocolized.

## References

[1] Terdiman JP, Ostroff JW (1998). Gastrointestinal bleeding in the hospitalized patient: a case-control study to assess risk factors, causes, and outcome. Am J Med.

[2] Czaja AJ, McAlhany JC, Pruitt BA Jr (1974). Acute gastroduodenal disease after thermal injury — an endoscopic evaluation of incidence and natural history. N Engl J Med.

[3] Moody FG, Cheung LY (1976). Stress ulcers: their pathogenesis, diagnosis, and treatment. Surg Clin North Am.

[4] Eisa N, Bazerbachi F, Alraiyes AH, Alraies MC (2014). Do all hospitalized patients need stress ulcer prophylaxis?. Cleve Clin J Med.

[5] Buendgens L, Koch A, Tacke F (2016). Prevention of stress-related ulcer bleeding at the intensive care unit: risks and benefits of stress ulcer prophylaxis. World J Crit Care Med.

[6] Geus WP, Lamers CB (1990). Prevention of stress ulcer bleeding: a review. Scand J Gastroenterol Suppl.

[7] Navab F, Steingrub J (1995). Stress ulcer: is routine prophylaxis necessary?. Am J Gastroenterol.

[8] Stremple JF, Molot MD, McNamara JJ, Mori H, Glass GB (1972). Posttraumatic gastric bleeding: prospective gastric secretion composition. Arch Surg.

[9] Bowen JC, Fleming WH, Thompson JC (1974). Increased gastrin release following penetrating central nervous system injury. Surgery.

[10] (1999). ASHP therapeutic guidelines on stress ulcer prophylaxis. Am J Health Syst Pharm.

[11] Cook DJ, Fuller HD, Guyatt GH (1994). Risk factors for gastrointestinal bleeding in critically ill patients. N Engl J Med.

[12] DePriest JL (1995). Stress ulcer prophylaxis. Postgrad Med.

[13] Malhis A, Alghamdi T, Alfandi R (2019). Appropriateness of acid-suppressing agents for stress ulcer prophylaxis in non-intensive care unit setting in Saudi Arabia. J Pharm Bioallied Sci.

[14] Shuman RB, Schuster DP, Zuckerman GR (1987). Prophylactic therapy for stress ulcer bleeding: a reappraisal. Ann Intern Med.

[15] Bardou M, Quenot JP, Barkun A (2015). Stress-related mucosal disease in the critically ill patient. Nat Rev Gastroenterol Hepatol.

[16] Horsa BA, Ayele Y, Ayalew MB (2019). Assessment of pharmacologic prophylaxis use against stress ulcer in the medical wards of University of Gondar Hospital. SAGE Open Med.

[17] Herzig SJ, Vaughn BP, Howell MD, Ngo LH, Marcantonio ER (2011). Acid-suppressive medication use and the risk for nosocomial gastrointestinal tract bleeding. Arch Intern Med.

[18] Qadeer MA, Richter JE, Brotman DJ (2006). Hospital-acquired gastrointestinal bleeding outside the critical care unit: risk factors, role of acid suppression, and endoscopy findings. J Hosp Med.

[19] Cook DJ (1995). Stress ulcer prophylaxis: gastrointestinal bleeding and nosocomial pneumonia best evidence synthesis. Scand J Gastroenterol Suppl.

[20] Cook DJ, Reeve BK, Guyatt GH, Heyland DK, Griffith LE, Buckingham L, Tryba M (1996). Stress ulcer prophylaxis in critically ill patients. Resolving discordant meta-analyses. JAMA.

[21] Reynolds PM, MacLaren R (2019). Re-evaluating the utility of stress ulcer prophylaxis in the critically ill patient: a clinical scenario-based meta-analysis. Pharmacotherapy.

[22] Finkenstedt A, Berger MM, Joannidis M (2020). Stress ulcer prophylaxis: is mortality a useful endpoint?. Intensive Care Med.

[23] Ogasawara O, Kojima T, Miyazu M, Sobue K (2020). Impact of the stress ulcer prophylactic protocol on reducing the unnecessary administration of stress ulcer medications and gastrointestinal bleeding: a single-center, retrospective pre-post study. J Intensive Care.

[24] Heidelbaugh JJ, Kim AH, Chang R, Walker PC (2012). Overutilization of proton-pump inhibitors: what the clinician needs to know. Therap Adv Gastroenterol.

[25] Ramos GF, Luglio M, Brunow de Carvalho W, Delgado AF (2020). Stress ulcer prophylaxis remains a controversial management in the PICU. Pediatr Crit Care Med.

[26] Deshpande A, Pasupuleti V, Thota P (2013). Acid-suppressive therapy is associated with spontaneous bacterial peritonitis in cirrhotic patients: a meta-analysis. J Gastroenterol Hepatol.

[27] Buckley MS, Park AS, Anderson CS (2015). Impact of a clinical pharmacist stress ulcer prophylaxis management program on inappropriate use in hospitalized patients. Am J Med.

[28] Langan RC, Goodbred AJ (2017). Vitamin B12 deficiency: recognition and management. Am Fam Physician.

[29] Cai D, Feng W, Jiang Q (2015). Acid-suppressive medications and risk of fracture: an updated meta-analysis. Int J Clin Exp Med.

[30] Thong BK, Ima-Nirwana S, Chin KY (2019). Proton pump inhibitors and fracture risk: a review of current evidence and mechanisms involved. Int J Environ Res Public Health.

[31] Farina C, Gagliardi S (2002). Selective inhibition of osteoclast vacuolar H(+)-ATPase. Curr Pharm Des.

[32] Barletta JF, Bruno JJ, Buckley MS, Cook DJ (2016). Stress ulcer prophylaxis. Crit Care Med.

[33] Heidelbaugh JJ, Inadomi JM (2006). Magnitude and economic impact of inappropriate use of stress ulcer prophylaxis in non-ICU hospitalized patients. Am J Gastroenterol.

[34] Franchitti M, Piubellini J, Sadeghipour F, Eckert P, Voirol P, Schneider AG (2020). Adequacy of stress ulcer prophylaxis prescription in the intensive care unit: an observational study. Swiss Med Wkly.

[35] Clarke K, Adler N, Agrawal D (2021). Reducing overuse of proton pump inhibitors for stress ulcer prophylaxis and nonvariceal gastrointestinal bleeding in the hospital: a narrative review and implementation guide. J Hosp Med.

[36] Biyase N, Perrie H, Scribante J, Muteba M, Chetty S (2021). Stress ulcer prophylaxis use in critical care units at public hospitals in Johannesburg, South Africa. South Afr J Crit Care.

[37] Xing XX, Zhu C, Chu YQ, Bai XR, Wang K, Zhang ST, Yan SY (2021). Physicians' knowledge, attitude, and prescribing behavior regarding stress ulcer prophylaxis in China: a multi-center study. BMC Gastroenterol.

[38] Wijaya D, Padolo E, Ardianto C, Matulatan F, Alderman CP (2020). Analysis of the use and cost of stress ulcer prophylaxis for surgical inpatients. J Basic Clin Physiol Pharmacol.

[39] Alagha H, Mohammed Al Mabhouh N (2022). Evaluation of current practice of stress ulcer prophylaxis in patients admitted to intensive care units in the gaza strip- palestine. Bulletin of Pharmaceutical Sciences Assiut.

[40] Shahbazi Khamas S, Darvishi F, Taghvaye-Masoumi H, Rafiei E, Jafari A (2022). The utilization evaluation of intravenous pantoprazole for stress ulcer prophylaxis in a major teaching hospital. J Pharm Care.

[41] Shahbazi F, Karimpur H, Hosseini E (2019). Implementation of stress ulcer prophylaxis (SUP) in an intensive care unit (ICU). J Pharm Res Int.

